# Entomological monitoring data driving decision-making for appropriate and sustainable malaria vector control in Côte d’Ivoire

**DOI:** 10.1186/s12936-023-04439-z

**Published:** 2023-01-12

**Authors:** Bernard Loukou Kouassi, Constant Edi, Allassane Foungoye Ouattara, Armand Kouassi Ekra, Louise Golou Bellai, Janice Gouaméné, Yves Alain Kadio Kacou, Jackson Koffi Ives Kouamé, Armel-Hermann Obo Béké, Firmain N’Dri Yokoli, Constant Guy N’Guessan Gbalegba, Emmanuel Tia, Roseline Monsan Yapo, Lucien Yao Konan, Roméo N’Tamon N’Tamon, Maurice Adja Akré, Alphonsine Amanan Koffi, Antoine Mea Tanoh, Pascal Zinzindohoué, Blaise Kouadio, Patricia L. Yepassis-Zembrou, Allison Belemvire, Seth R. Irish, Ndombour Gning Cissé, Cecilia Flatley, Joseph Chabi

**Affiliations:** 1 PMI VectorLink project, Abidjan, Côte d’Ivoire; 2 Swiss Centre of Scientific Research in Côte d’Ivoire, Abidjan, Côte d’Ivoire; 3 Centre of Veterinary and Medical Entomology, Abidjan, Côte d’Ivoire; 4 National Institute of Public Hygiene, Abidjan, Côte d’Ivoire; 5 National Institute of Public Health/Pierre Richet Institute, Bouake, Côte d’Ivoire; 6 National Malaria Control Programme, Abidjan, Côte d’Ivoire; 7 U.S. President’s Malaria Initiative, USAID, Abidjan, Côte d’Ivoire; 8 U.S. President’s Malaria Initiative, Centers for Disease Control and Prevention (CDC), Abidjan, Côte d’Ivoire; 9 U.S. President’s Malaria Initiative, USAID, Washington, DC USA; 10 U.S. President’s Malaria Initiative, Entomology Branch, U.S. Centers for Disease Control and Prevention (CDC), Atlanta, GA USA; 11 PMI VectorLink Project, Washington, DC USA

**Keywords:** Malaria, *Anopheles gambiae*, Insecticide resistance monitoring, Vector bionomics, ITNs, IRS, Decision-making, Côte d’Ivoire

## Abstract

**Background:**

Entomological surveillance provides critical information on vectors for appropriate malaria vector control and strategic decision-making. The widely documented insecticide resistance of malaria vectors in Côte d’Ivoire requires that any vector control intervention deployment be driven by entomological data to optimize its effectiveness and appropriate resource allocations.

To achieve this goal, this study documents the results of monthly vector surveillance and insecticide susceptibility tests conducted in 2019 and a review of all previous entomological monitoring data used to guide vector control decision making. Furthermore, susceptibility to pirimiphos-methyl and clothianidin was assessed in addition to chlorfenapyr and pyrethroids (intensity and piperonyl butoxide (PBO) synergism) tests previously reported. Vector bionomic data were conducted monthly in four sites (Sakassou, Béoumi, Dabakala and Nassian) that were selected based on their reported high malaria incidence. Adult mosquitoes were collected using human landing catches (HLCs), pyrethrum spray catches (PSCs), and human-baited CDC light traps to assess vector density, behaviour, species composition and sporozoite infectivity.

**Results:**

Pirimiphos-methyl and clothianidin susceptibility was observed in 8 and 10 sites, respectively, while previous data reported chlorfenapyr (200 µg/bottle) susceptibility in 13 of the sites, high pyrethroid resistance intensity and increased mortality with PBO pre-exposure at all 17 tested sites.

*Anopheles gambiae *sensu lato was the predominant malaria vector collected in all four bionomic sites. Vector density was relatively higher in Sakassou throughout the year with mean biting rates of 278.2 bites per person per night (b/p/n) compared to Béoumi, Dabakala and Nassian (mean of 48.5, 81.4 and 26.6 b/p/n, respectively). The mean entomological inoculation rate (EIR) was 4.44 infective bites per person per night (ib/p/n) in Sakassou, 0.34 ib/p/n in Beoumi, 1.17 ib/p/n in Dabakala and 1.02 ib/p/n in Nassian. The highest EIRs were recorded in October in Béoumi (1.71 ib/p/n) and Nassian (3.22 ib/p/n), in July in Dabakala (4.46 ib/p/n) and in May in Sakassou (15.6 ib/p/n).

**Conclusion:**

Based on all results and data review, the National Malaria Control Programme developed and implemented a stratified insecticide-treated net (ITN) mass distribution in 2021 considering new generation ITNs. These results also supported the selection of clothianidin-based products and an optimal spraying time for the first indoor residual spraying (IRS) campaign in Sakassou and Nassian in 2020.

**Supplementary Information:**

The online version contains supplementary material available at 10.1186/s12936-023-04439-z.

## Background

Malaria is one of the most important diseases of poverty and its public health relevance, particularly in sub-Saharan Africa, is still a concern [1]. It remains a primary cause of illness and deaths worldwide, with 84 countries around the world reporting malaria cases and deaths in 2021 [2]. However, significant progress has been made over the past 20 years resulting in a reduction of malaria prevalence by 50%, with reports dropping to 232 million cases in 2019, though the recent COVID-19 pandemic has impacted the gain with increasing cases to 245 million and 247 million observed in 2020 and 2021 respectively. Nonetheless, there was a decreasing number of deaths worldwide due to malaria following reports of 619,000 deaths in 2021 compared to 625,000 deaths in 2020 [2].

Preventive measures are the most effective and commonly prioritized control methods recommended by the World Health Organization (WHO) for malaria control. Insecticide-based vector control still represents the main control method, which has enabled the reduction of malaria burden over the last two decades [1, 3]. Insecticide-treated nets (ITNs) and indoor residual spraying (IRS), represent the core vector control interventions for malaria prevention and their widespread use has resulted in significant decreases in malaria morbidity and mortality [1, 3]. Entomological surveillance is conducted to ensure optimal deployment of these interventions, and to guide insecticide resistance management strategies. Five “pillars” have been proposed by the WHO to support and orient malaria endemic countries on insecticide resistance management [4]. According to the WHO, all National Malaria Control Programme (NMCP) and stakeholder decisions must be strategically linked to entomological surveillance activities to inform vector control planning and implementation and to ensure that appropriate interventions are being used where they should be.

A greater emphasis was put on strengthening national capacities to generate, analyse and use high quality entomological surveillance data to maximize impact of vector control strategies [5]. Furthermore, the effectiveness of vector control interventions depends on accurate information regarding distribution and abundance of the main vector species and their resistance to insecticides [6–10], and this data can only be produced through entomological monitoring. In most endemic countries, the spread of vectors resistant to the insecticides used in ITNs and IRS has been widely reported as the outcomes of long-term use of the same class of insecticide in public health vector control [6–10] in addition to the unregulated use of pesticides in agriculture [11, 12]. This requires timely and close monitoring of the vectors following WHO recommendations whilst new insecticides and products are becoming available.

Furthermore, potential malaria transmission within a country is often heterogeneous and differs significantly from one location to another due to the eco-geographical position of sites. Important bionomics traits such as vector species composition, biting time, biting behaviour, host preference, and resting behaviours should be known for all the principal malaria vectors and assessed over time to detect any seasonal or other environmental changes that could affect the effectiveness of interventions [13].

In Côte d’Ivoire, malaria remains a major public health problem with a general population incidence of 441 malaria cases per thousand inhabitants and morbidity of about 173 deaths per thousand in 2020 among the children under 5 years old [14]. *Anopheles gambiae *sensu lato (*s.l*.), *Anopheles funestus s.l.* and *Anopheles nili* are the three major malaria vectors in the country [15, 16], with *An. gambiae s.l.* being the main vector across the country. The annual entomological inoculation rate (EIR) was also heterogeneous across the country and ranged between 6 and 789 *Plasmodium falciparum*-infected bites per person per year [15, 17, 18]. *Plasmodium falciparum* was responsible for about 99% of the uncomplicated and severe malaria cases, followed by *Plasmodium malariae* [14].

The country is known as one of the areas hosting highly resistant malaria vector populations. This has been confirmed in a section of this project by Kouassi et al. [19], reporting the resistance status and intensity of *An. gambiae s.l.* in about 15 sites selected across the country. Furthermore, previous studies had also highlighted the same trends in different parts of the Côte d’Ivoire, with several resistance mechanisms involved in the resistance of the vector populations [20–24].

This level of resistance was certainly affecting efforts that the country was making to increase the access to insecticide-based vector control interventions. Current strategies for malaria vector control in Côte d’Ivoire are universal mass ITN distribution every three years, routine distribution of ITNs to pregnant women during their first antenatal care visit, to children under one year of age during immunization visits and to children between one and five years of age during health consultations. It is important to note that only pyrethroid-only ITNs had been distributed in Côte d’Ivoire prior to 2021, and no efforts were made to correlate ITN distributions and malaria vector resistance in each of the recipient districts, while the effectiveness of vector control using pyrethroid treated tools is now being threatened by the spread of insecticide resistance, coupled with vector behaviour such as increase in outdoor biting or daytime biting [6, 7, 25, 26].

Supported by the U.S. President’s Malaria Initiative (PMI), and by the Global Fund, the NMCP of Côte d’Ivoire through its 2016–2020 malaria national strategic plan (NSP) opted for new approaches to malaria control [27]. Therefore, the NMCP decided, based on entomological evidence, to introduce new types of ITNs incorporating either synergist or dual active ingredients for mass and routine distribution and implement targeted IRS within the country. Due to the widely reported insecticide resistance of the vectors and the heterogeneity of malaria endemicity in Côte d’Ivoire, it was important to use entomological data for intervention decision making to ensure appropriate allocation of resources and optimize the effectiveness of these interventions and ultimately increase impact on malaria vectors.

This study presents entomological baseline data collections including monthly vector surveillance and annual insecticide susceptibility tests conducted in 2019 in addition to literature review of all previous entomological monitoring data to guide ITN selection for mass distribution and IRS implementation within the country in 2020.

## Methods

### Study sites

**Fig. 1:**
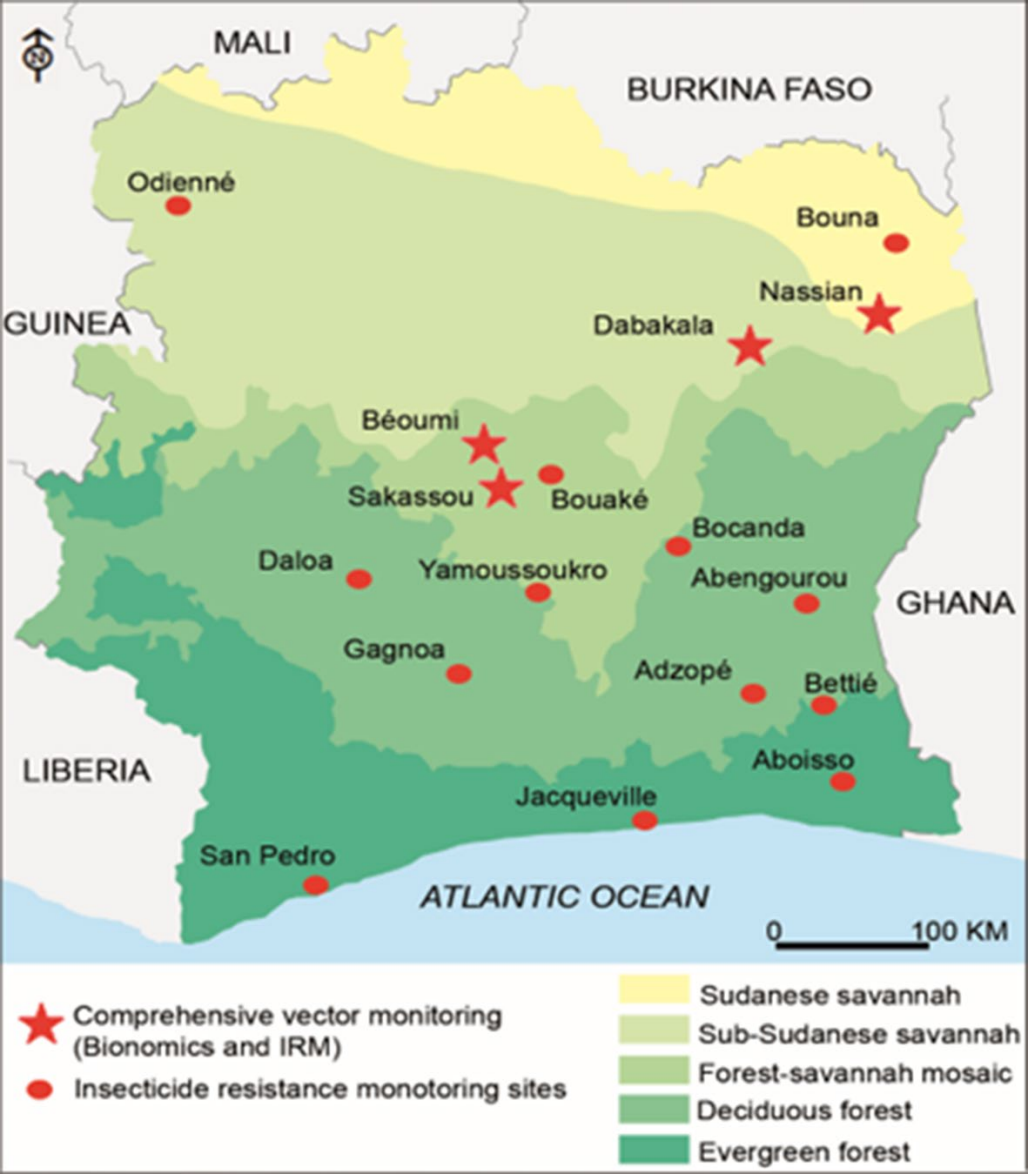
Map of the entomological monitoring sites

Côte d’Ivoire is a coastal country divided into five different geographical zones including an evergreen forest zone in the south and costal part of the country, a deciduous forest zone, a forest/savanna mosaic zone in the centre considered as a transitional zone between the South and the North, a sub-Sudanese savannah zone, and a Sudanese savannah zone both in the northern part of the country. The climate in the South is equatorial, with annual rainfall between 2100 mm and 2500 mm and sub-equatorial in the western forest with average rainfall between 1600 mm and 2300 mm per year. In the central transitional zone, the climate is tropical, with an average of 1200 mm rainfall per year. The climate is also tropical in the northern sub-Sudan and Sudan zones, with an annual average rainfall of 900 mm [Société d’Exploitation et de Développement Aéroportuaire, Aéronautique et Météorologique (SODEXAM)]. Insecticide resistance data review was conducted in a total of 17 sites distributed across the country including the 15 sites reported by Kouassi et al. in 2020 [16] and the sites of Bocanda and Jacqueville where data was available. The longitudinal monitoring surveys were carried out in four of these sites including Nassian, Sakassou Beoumi and Dabakala (Fig. 1). Four houses were selected in each district to serve as mosquito collection houses and used throughout the collection period.

### WHO insecticide susceptibility tests

Insecticide susceptibility tests were conducted between June and September 2019 on 2–5-day old adult female *An. gambiae s.l.* reared from field collected larvae from each of the surveyed site. Various larval habitats were surveyed within each of the sites and, collected larvae were pooled and reared to adults at a field laboratory. The resistance status of the local vectors against the diagnostic concentrations of permethrin (0.75%), deltamethrin (0.05%), alpha-cypermethrin (0.05%) and pirimiphos-methyl (0.25%) was determined using WHO tube tests [28]. When resistance was confirmed, the intensity of resistance at 5 and 10 times the diagnostic concentration of each insecticide and synergism using 4% piperonyl butoxide (PBO) with one hour pre-exposure and pyrethroids, were conducted to assess the level of resistance and implication of mono-oxygenase enzymes using WHO tube tests [28].

Additionally, clothianidin papers were treated locally at a dose of 2% using 264 mg of a formulated SumiShield product diluted in 20 ml of distilled water. Two millilitres of the solution were used to impregnate a 12 × 15 cm Whatman filter paper grade 1. The treated papers were dried overnight and preserved in aluminum foil at 4˚C before testing is conducted. The susceptibility testing was conducted as described above, and the mortality was recorded every 24 h, up to seven days post-exposure. CDC bottle assays were used to evaluate the susceptibility of the vectors to chlorfenapyr at two doses (100 µg a.i/bottle and 200 µg a.i/bottle when resistance was observed to 100 µg a.i/bottle) with mortality recorded up to 72 h [29]. Testing conditions were recorded at the start and end of the test with an average temperature of 25ºC (± 2 ºC).

### Adult mosquito collection

Adult mosquitoes were collected monthly from January to December 2019 in Sakassou (12 months) and from May to December 2019 (8 months) in Nassian, Dabakala and Beoumi, using human landing catches (HLC), pyrethrum spraying catches (PSC), and CDC light trap collection methods. The longitudinal monitoring started later in Nassian, Dabakala, and Beoumi compared to Sakassou due to change of initially selected sites which included Gagnoa, Jacqueville, and Bocanda.

HLCs were conducted indoors and outdoors in four houses per site during two consecutive nights per month to collect adult mosquitoes landing on human baits. The human-baited CDC light trap collections were performed in four different houses from HLCs during two consecutive nights per site per month. CDC light traps were suspended indoors where people slept under a treated ITN (received from mass ITN distribution campaign) from 6:00 p.m. to 6:00 a.m. The PSCs were conducted in 30 houses per site per month between 6:00 a.m. and 8:00 a.m. For houses with open eaves, collectors sprayed from the outside through the eaves before entering and spraying indoors and all mosquitoes knocked down were collected from the white sheets and placed into Petri dishes. The same houses were maintained for HLC and CDC light trap collections throughout the longitudinal monitoring period, while PSC collections were conducted in randomly selected houses, depending on the availability of households. All *Anopheles* mosquitoes collected were morphologically identified using the key of Gillies and Coetzee [30] and the potential vectors such as *An. gambiae s.l*., *An. funestus s.l*. and *An. nili* were individually preserved in Eppendorf tubes containing silica gel desiccant for further laboratory analyses.

### Species identification and sporozoite infection detection of malaria vectors

In the four longitudinal monitoring sites, a subsample of about 80 samples of the preserved vectors was randomly selected per month to determine subspecies of *An. gambiae s.l.* and *P. falciparum* sporozoite infection. The DNA of each individual mosquito was extracted using the protocol designed by Collins et al. [31]. *Anopheles gambiae* complex species were identified following the Short-Interspersed Element protocol described by Santolamazza et al. [32]. The sporozoite infection of a subsample of *An. gambiae s.l.* and *An. funestus s.l.* collected by HLC from each site was determined using the enzyme-linked immunosorbent assay protocol for identification of *P. falciparum* circumsporozoite infection [33] and all positive mosquitoes were retested for confirmation.

### Malaria epidemiological data of Sakassou and Nassian

To overlap the entomological monitoring data and the epidemiological data over the year for a more robust decision-making process, the number of rapid diagnostics tested (RDT) malaria cases from the previous two years (2017 and 2018) were collected through the country health information management system (HIMS) data as monthly reported cases to provide estimates of the number of confirmed cases and periodicity throughout the year to estimate the peak of malaria incidences at each site. However, the 2019 HMIS data was not available during the decision-making time.

### Statistical analysis

Insecticide resistance status was defined following WHO criteria [28] measuring mortality after 24 h for pyrethroids and pirimiphos-methyl, after 3 days for chlorfenapyr and 7 days for clothianidin. Mortality of less than 90% was considered as “confirmed resistance”, between 90 and 98% as “possible resistance”, and 98% and greater as “susceptible”. The mean human biting rate (HBR) and entomological inoculation rate (EIR) was calculated using the average of all collection-months per site. The Kruskal–Wallis equality of population rank test was used to compare the mean HBR and EIR between the four different sites. All differences were considered significant at P < 0.05.

## Results

### WHO susceptibility test, intensity, and synergist assays

Kouassi et al. [19] had already reported high pyrethroid resistance at 15 sites tested and a substantial increase in vector mortality after pre-exposure to PBO. In addition to those sites, high resistance intensity was also recorded against pyrethroid insecticides in Bocanda and Jacqueville, and pre-exposure to PBO increased vector mortality when exposed to deltamethrin, alpha-cypermethrin, and permethrin as observed in the other reported sites [19].

### Susceptibility status of An. gambiae to clothianidin and pirimiphos-methyl

**Fig. 2:**
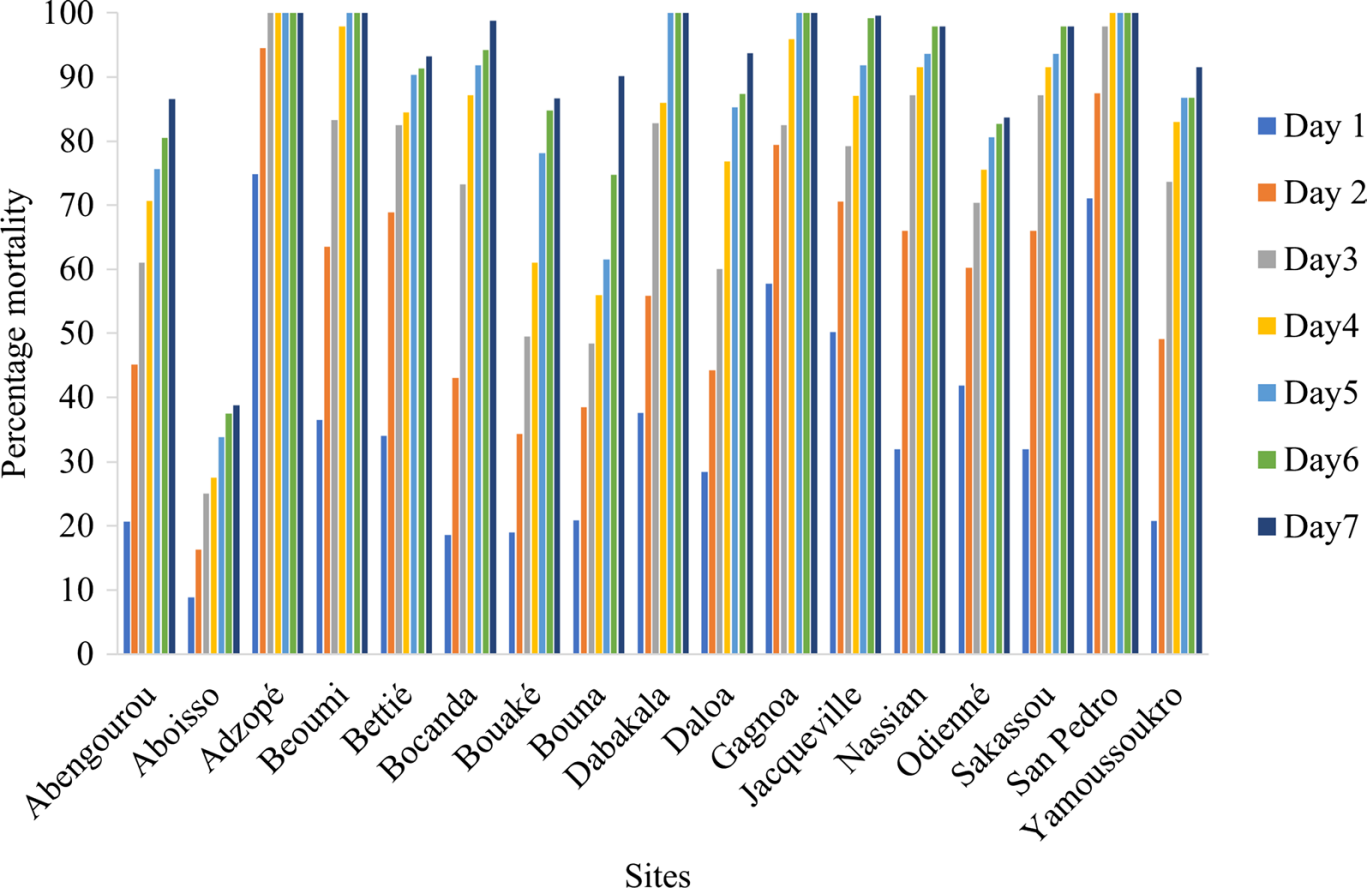
Susceptibility of *An. gambiae s.l.* to pirimiphos-methyl by site in 2019

**Fig. 3:**
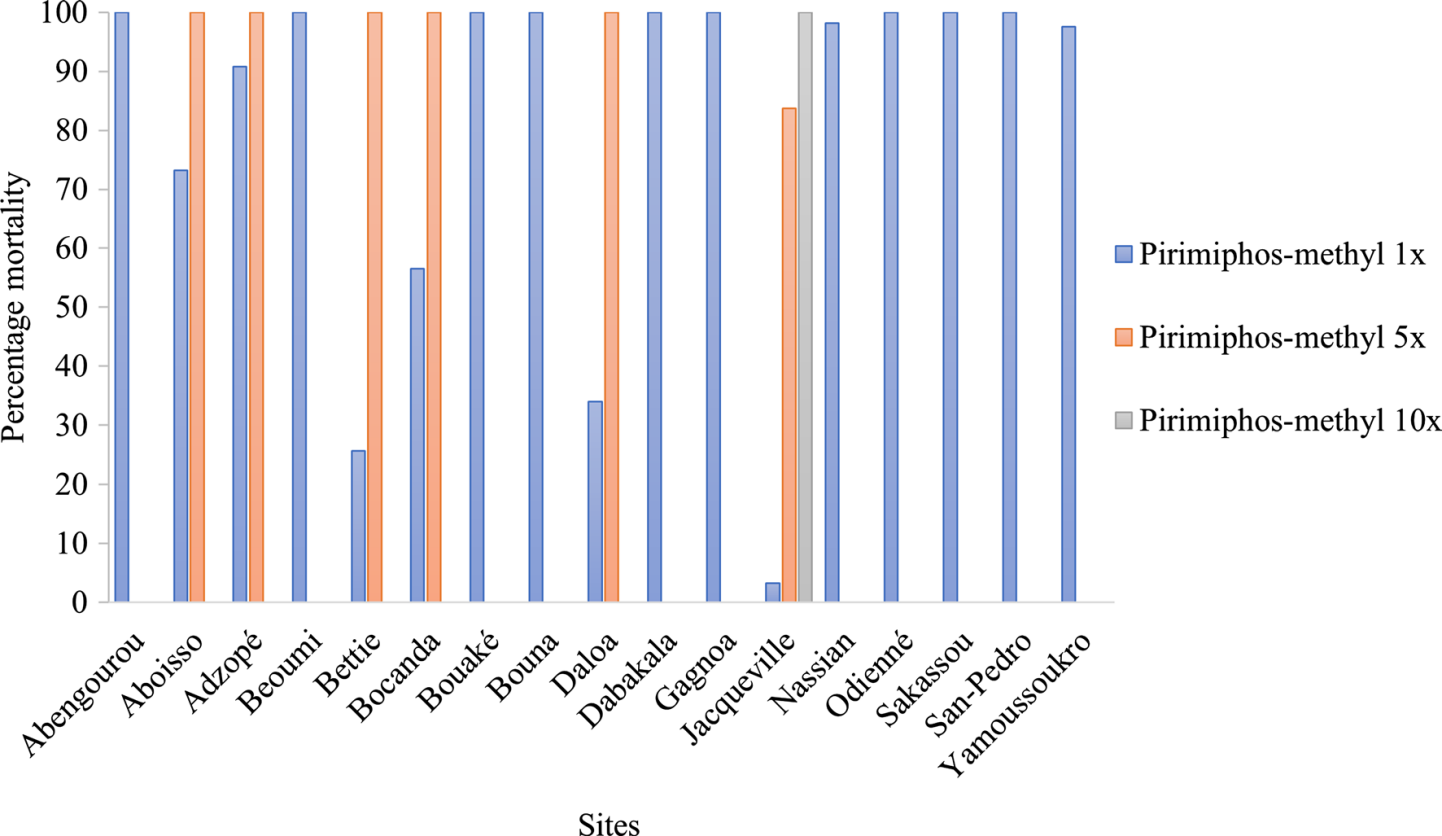
Susceptibility of *An. gambiae s.l.* to clothianidin 2% by site in 2019

For clothianidin, susceptibility was recorded in 9 out of the 17 sites including Nassian and Sakassou, both initially targeted districts for IRS. Resistance to clothianidin was observed in Abengourou, Aboisso, Bettié, Bouaké, Bouna, Daloa, Odienné, and Yamoussoukro (Fig. 2). Susceptibility to pirimiphos-methyl was recorded at the diagnostic dose in 11 sites, while low resistance intensity was observed in 5 of the remaining sites (Aboisso, Adzopé, Bettié, Bocanda, and Daloa). Only Jacqueville recorded a moderate resistance to pirimiphos-methyl (Fig. 3).

### Adult mosquito fauna across the four bionomic monitoring sites

A total of 105,225 mosquitoes, including 12,206 (11.6%) culicines and 93,019 *Anopheles* (88.4%) were collected over the year (January through December 2019) using the three collection methods (HLC, PSC and CDC light trap). *Anopheles gambiae s.l.* was the predominant malaria vector species, representing 94.4% (87,765/93,019) of the total *Anopheles* mosquitoes collected across all sites.

Using HLC, a total of 77,122 *Anopheles* mosquitoes were collected. With PSC and CDC light trap methods, 10,254 and 5,643 mosquitoes were collected, respectively. *Anopheles gambiae s.l.* was the most collected vector using all three methods (Table 1).

**Table 1:** Number and percentages of mosquitoes collected in all sites using the three collection methods in 2019

Collection method	Species	Béoumi# (%)	Dabakala# (%)	Nassian# (%)	Sakassou# (%)	Total# (%)
All collection methods	*An. gambiae s.l*	7,930 (90.6)	14,864 (88.8)	3,968 (91.4)	61,003 (96.5)	87,765 (94.4)
	*An. funestus s.l*	7 (0.1)	998 (6.0)	368 (8.5)	282 (0.45)	1,655 (1.8)
	*An. nili*	1 (0.2)	560 (3.4)	0 (0)	13 (0.1)	574 (0.6)
	^a^Other *Anopheles*	819 (27.1)	310 (10.2)	7 (0.2)	1,889 (62.4)	3,025 (3.3)
	**Total**	**8757**	**16732**	**4343**	**63,187**	**93,019**
HLC	*An. gambiae s.l*	6,102 (88.5)	10,643 (92.7)	3,408 (92.5)	53,179 (96.5)	73,332 (95.1)
	*An. funestus s.l*	7 (0.1)	281 (2.4)	270 (7.3)	159 (0.3)	717 (0.9)
	*An. nili*	1 (0.0)	426 (3.7)	0 (0)	13 (0.0)	440 (0.6)
	^*^Other *Anopheles*	783 (11.4)	135 (1.2)	7 (0.2)	1708 (3.1)	2633 (3.4)
	**Total**	**6893**	**11,485**	**3685**	**55,059**	**77,122**
PSC	*An. gambiae s.l*	1,183 (97.0)	2,381 (69.9)	501 (83.6)	4,725 (94.0)	8,790 (85.7)
	*An. funestus s.l*	0 (0)	717 (21.0)	98 (16.4)	123 (2.4)	938 (9.1)
	*An. nili*	0 (0)	134 (3.9)	0 (0)	0 (0)	134 (1.3)
	^*^Other *Anopheles*	36 (3)	175 (5.1)	0 (0)	181 (3.6)	392 (3.8)
	**Total**	**1219**	**3407**	**599**	**5,029**	**10,254**
CDC light trap	*An. gambiae s.l*	645 (100)	1840 (100)	59 (100)	3099 (100)	5643 (100)
	**Total**	**645**	**1840**	**59**	**3099**	**5643**

^*^Other* Anopheles * included *Anopheles pharoensis*, *Anopheles ziemanni* and *Anopheles coustani*; rows in bold represent the total Anopheles collected per site

Out of the 93,019 *Anopheles*, 8,757 were collected in Béoumi, 16,732 in Dabakala, 4,343 in Nassian from May through December and 63,187 in Sakassou from January through December 2019. *Anopheles gambiae s.l.* was the most collected malaria vector species in the four sites representing 90.6% (n = 7,930), 88.8% (n = 14,864), 91.4% (n = 3,968) and 96.5% (n = 61,003) of the total *Anopheles* collected in Béoumi, Dabakala, Nassian and Sakassou, respectively. *Anopheles funestus s.l.* was the other malaria vector found in Beoumi (0.1%; n = 7) and Nassian (8.5%; n = 368), while both *An. funestus s.l.* and *An. nili* were found in Dabakala (6%; n = 998 and 3.4%; n = 560), and Sakassou (0.45%; n = 282 and 0.1%, n = 13), respectively (Table 1).

### Molecular species identification of adults An. gambiae collected for bionomic monitoring

**Fig. 4:**
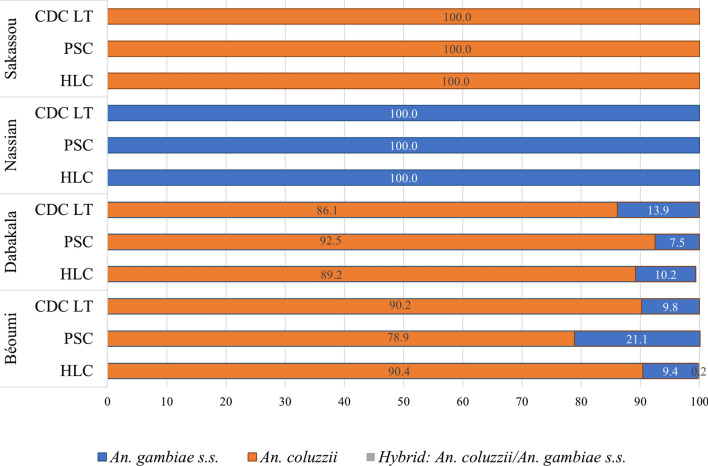
Proportion of *An. gambiae* species per collection methods in the bionomic monitoring sites in 2019

A subsample of 694 *An. gambiae s.l.* from Béoumi, 698 from Dabakala, 608 from Nassian, and 731 from Sakassou were molecularly identified to the sub-species level. *Anopheles coluzzii* represented the predominant species in Béoumi (mean of 86.5%) and Dabakala (mean of 89.3%) (all collection methods included) and was the only species found in Sakassou. In Nassian, *An. gambiae *sensu stricto (*s.s*.) was the only vector species found using the three methods. Few hybrids of the two species were found in the HLC samples from Béoumi (0.2%) (Fig. 4).

### Anopheles gambiae human biting rate

**Fig. 5:**
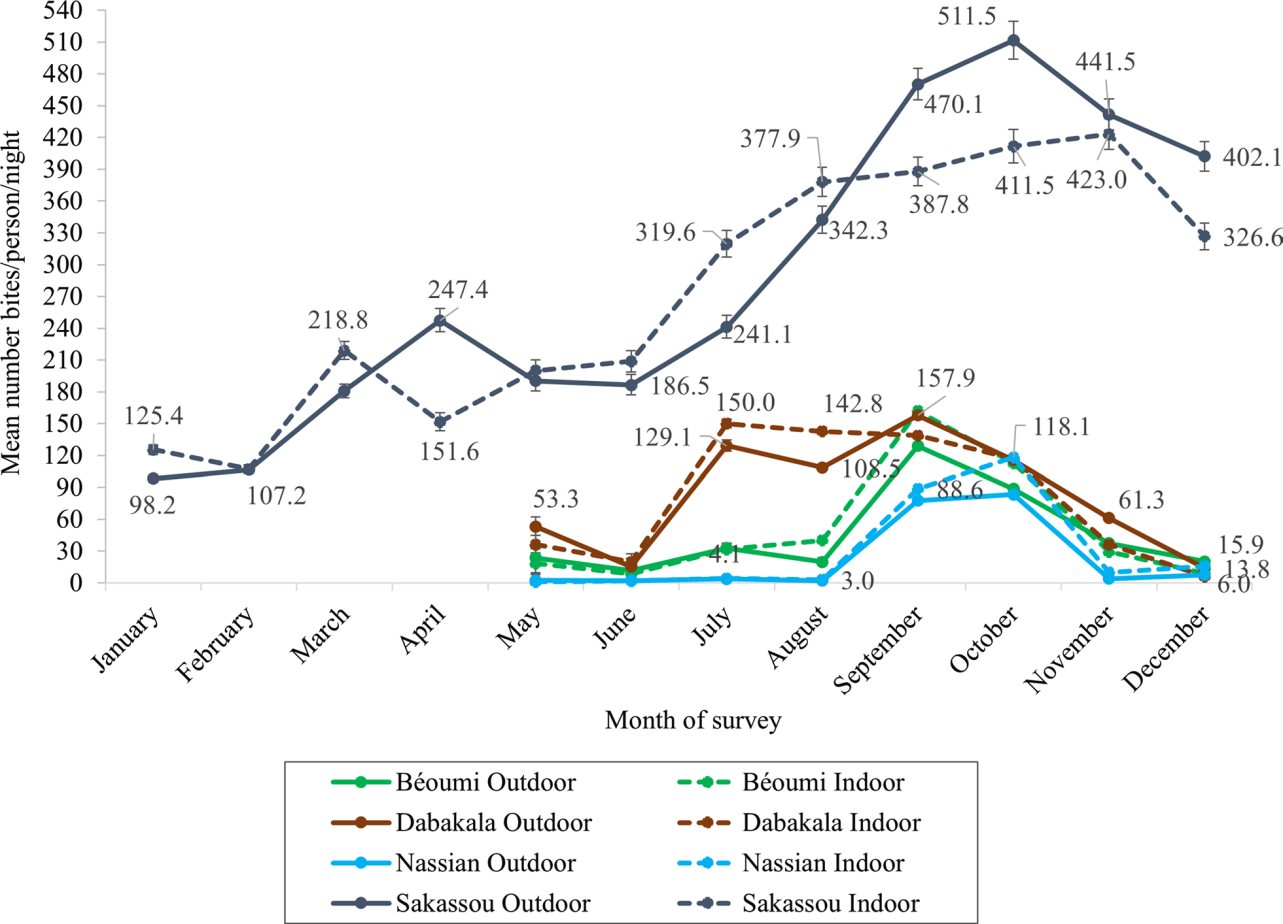
*Anopheles gambiae s.l.* biting rate using HLC at all sites in 2019

The human biting rates (HBRs) were higher in Sakassou (*p* = 0.0001), with an overall rate ranging from 125.4 b/p/n in January to 423.0 b/p/n in November indoors and from 98.2 b/p/n in January to 511.6 b/p/n in October outdoors compared to Dabakala (6.0 b/p/n in December to 150.0 b/p/n in July indoors and 13.8 b/p/n in December to 157.8 b/p/n in September outdoors) and Béoumi (9.2 b/p/n in December to 161.8 b/p/n in September indoors and 11.6 b/p/n in June to 128.8 b/p/n in September outdoors b/p/n). The mean biting rates were lowest in Nassian (from 0.8 b/p/n in May to 118.2 b/p/n in October indoors and 2.2 b/p/n in August to 83.4 b/p/n in October outdoors) regardless of the months and the collection place (indoor or outdoor) (Fig. 5). Overall, *An. gambiae s.l.* showed similar biting behaviour across the four districts with peak biting time observed between 10:00 p.m-3:00 a.m. (Additional file 1). However, *An. gambiae s.l.* bite slightly higher, but not significantly different outdoors (51.2%) in Sakassou than indoors, and indoors than outdoors in Béoumi (53.2% indoors) and Nassian (56.9% indoors), while equal percentages were recorded both outdoors and indoors (50%) in Dabakala.

### Plasmodium falciparum sporozoite infection and entomological inoculation rate

Table 2 shows the overall infection rate and EIRs for *An. gambiae s.l.* and *An. funestus s.l.* collected by HLC in the four districts. Nassian recorded the highest *P. falciparum* sporozoite infection rate (SR) among *An. gambiae s.l.* (0.055), followed by Sakassou (0.021) and Dabakala (0.018). The lowest rate (0.010) was recorded in Beoumi. Additionally, 16 *An. funestus s.l.* mosquitoes were found with sporozoite infections among the samples collected in November and December 2019, representing a SR of 7.4% of the 215 analysed mosquitoes in Nassian.

**Table 2:** Sporozoite infection rate and EIR of malaria vectors collected using HLC in the four vector bionomics sites

District	*An. gambiae s.l*						*An. funestus s.l*					
	TC	TA	P	Mean SR	Mean HBR(b/p/n)	Mean EIR(ib/p/n)	TC	TA	P	Mean SR	Mean HBR(b/p/n)	Mean EIR(ib/p/n)
Béoumi	7,930	900	9	0.011	48.47	0.34^c^	7	0	–	–	–	–
Dabakala	14,864	868	16	0.018	81.36	1.17^b^	998	0	–	–	–	–
Nassian	3,968	675	37	0.045	26.62	1.02^b^	368	215	16	0.074	16.4	1.21
^#^Sakassou	52,484	1541	25	0.018	278.2	4.44^a^	282	0	–	–	–	–

*TC* total collected*; TA* total analysed*; P* positive*; SR* sporozoite rate*; HBR* Human Biting Rate*; EIR* Entomological Inoculation Rate*; (*–*)* not analysed*. EIR* with different symbols are significantly different

^#^12 collection months were conducted in Sakassou and 8 months in the other 3 sites; the mean EIR of sites that have the same letter (a, b or c) are not significantly different

**Fig. 6:**
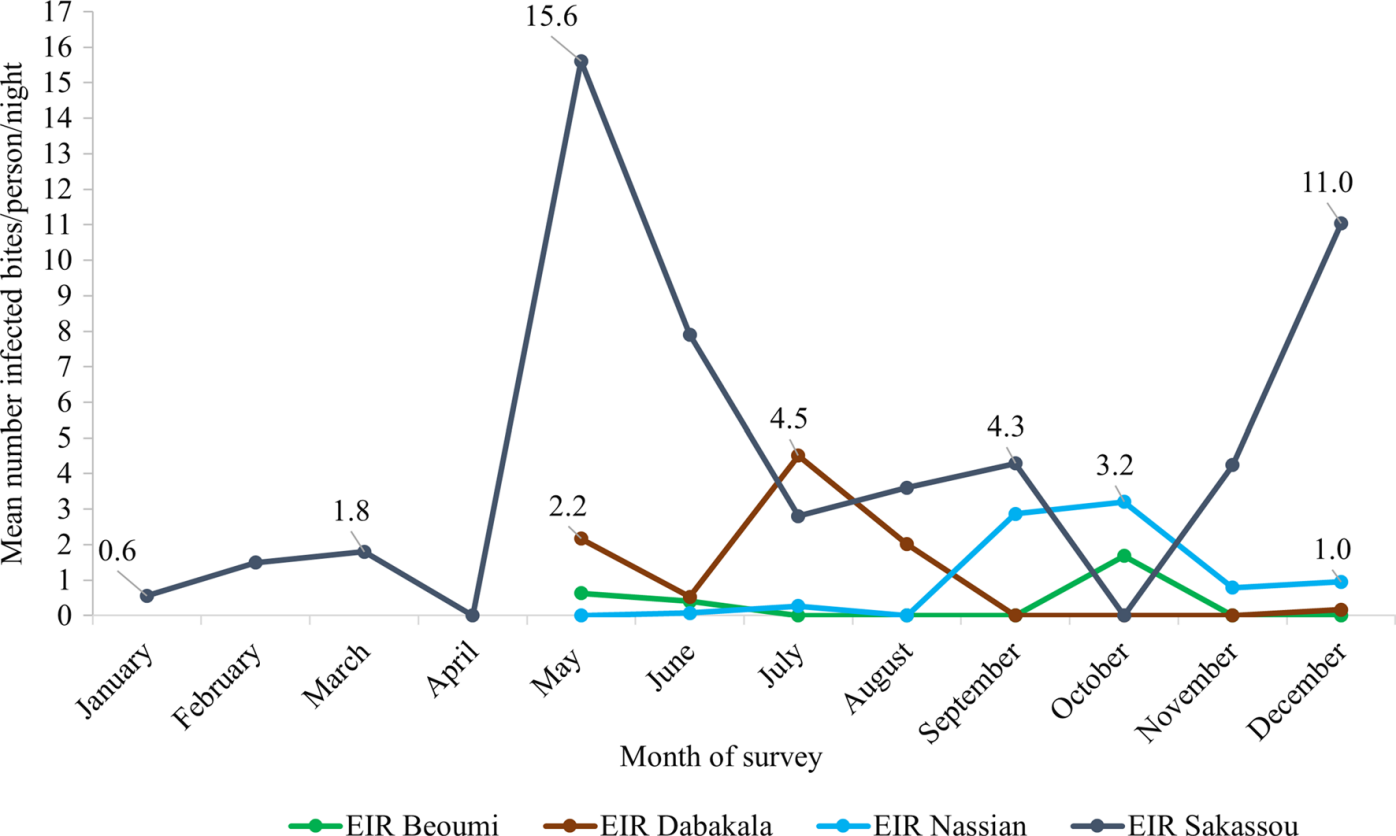
EIR of Malaria Vectors Collected Using HLC in the Four Vector Bionomics sites in 2019

Sakassou recorded the highest EIR (4.44 ib/p/n) of *An. gambiae* s.l. (*p* = 0.0272), while Nassian and Dabakala had similar EIRs of 1.02 and 1.17 ib/p/n, respectively. Béoumi recorded the lowest EIR among the four sites, with 0.34 ib/p/n. May and November were the months with the highest number of *An. gambiae s.l.* infected bites in Sakassou, while Dabakala recorded a single peak in July, Béoumi, and Nassian in October (Fig. 6). An average EIR value of 1.21 ib/p/n was recorded for *An. funestus s.l*. analysed in Nassian.

### Epidemiological data of targeted sites

HMIS data from 2017 and 2018 were averaged to provide monthly estimates of the number of confirmed cases within the population that were recorded from all health facilities of each district.

**Fig. 7:**
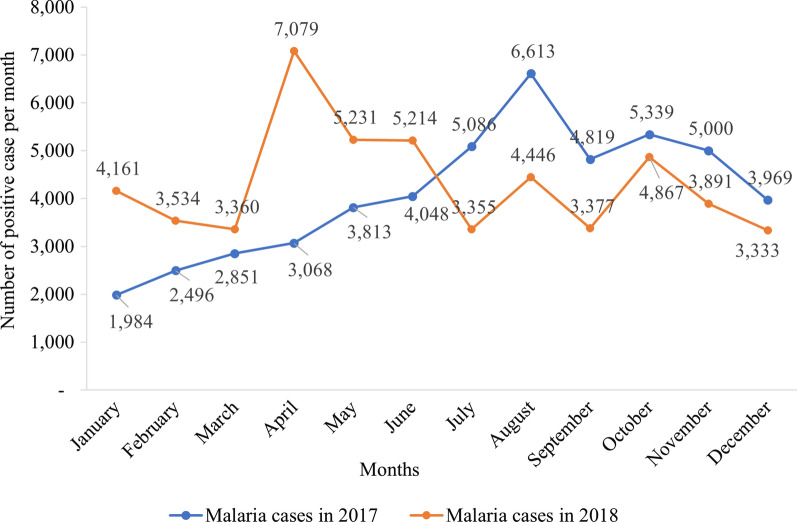
Malaria confirmed cases over time in Sakassou in 2017–2018

For Sakassou, a peak of number of positive malaria cases was observed in August 2017 and in April 2018. Malaria incidence was averagely stable in the health district between May and December 2017 (around 4000–5000 cases) except the peak of 6,613 cases in August, while all months had cases above 3000 from January to December 2018 with the peak of more than 7000 malaria cases in April 2018. The lowest number of malaria cases was recorded between January and March 2017 in Sakassou (Fig. 7).

**Fig. 8:**
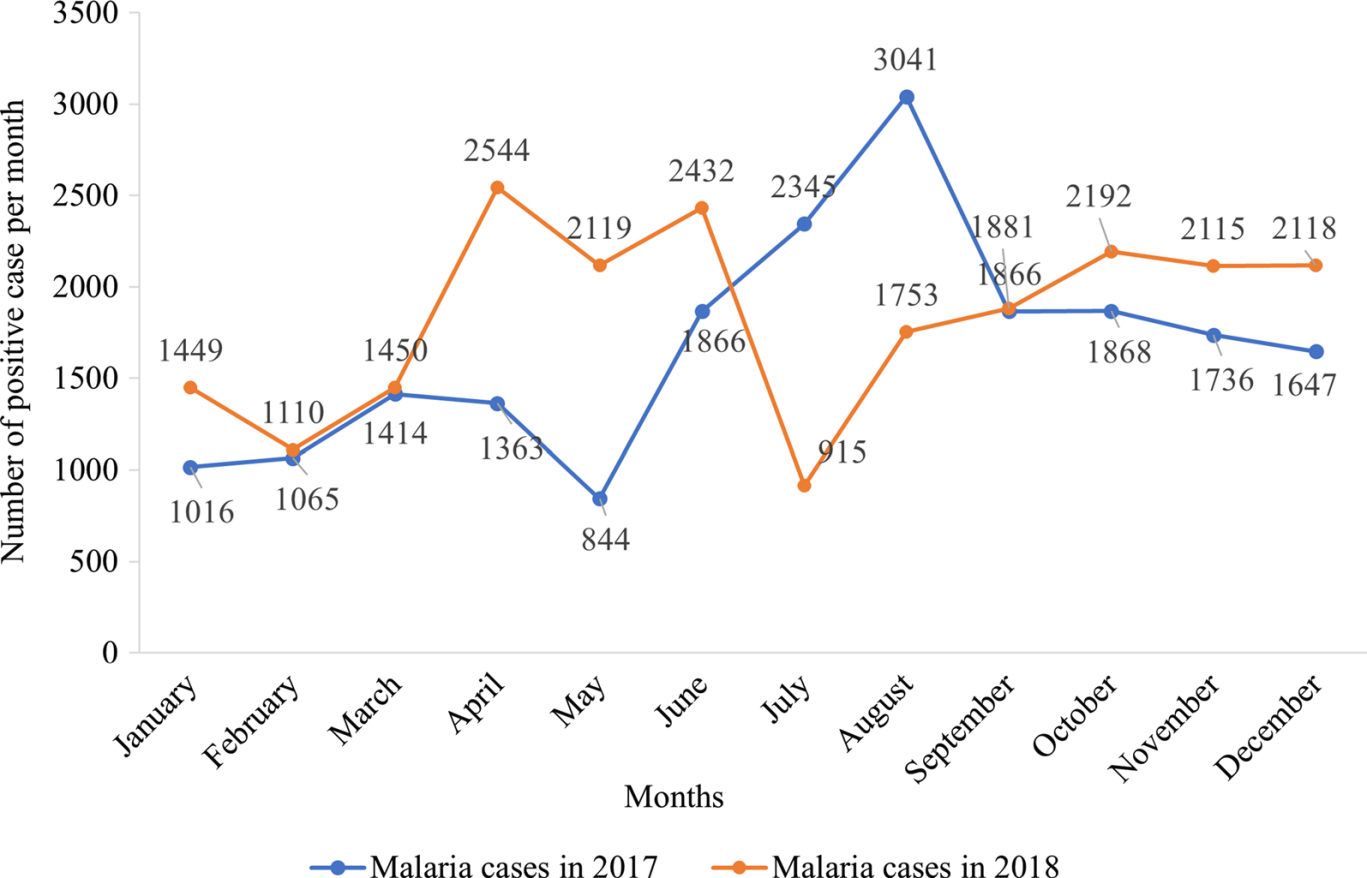
Malaria confirmed cases over time in Nassian in 2017–2018

In Nassian, even though the number of cases was slightly lower than Sakassou, an incidence peak was observed in August 2017 (3,041 malaria cases) while two similar peaks were recorded in April 2018 (2,544 cases) and in June 2018 (2,432 cases). However, the number of malaria cases were slightly higher in 2018 than 2017 except for a drop in cases that occurred in July 2018 (Fig. 8).

## Discussion

The study was set to collect and analyse entomological surveillance data to support and guide appropriate vector control decision-making in Côte d’Ivoire. To make good use of all the insecticide resistance monitoring data collected across the country and to support the NMCP’s decision making to achieve its national strategic plan (NSP) goals targeting the reduction of malaria cases by at least 75% in 2025 and pre-elimination by 2030, the NMCP, malaria stakeholders and partners prioritized the selection of ITNs for the 2021 mass distribution and, IRS district and insecticide selections to be based on data. For consensus, the vector control committee composed of the country’s entomologists, financial and technical partners, epidemiologists, and NMCP, conducted a systematic review and analysis of insecticide resistance monitoring data available to design a stratified ITN mass distribution which would allocate ITNs by district following the resistance status of local vectors. Similarity of pyrethroid resistance, resistance intensity and PBO synergism was found among all previously reported data by Kouassi et al. [19], and Bocanda and Jacqueville were two additional sites investigated [19].

Regarding the IRS to be implemented in the country, the vector surveillance data in all four study sites showed that, the proportion of vectors caught outdoors and indoors using HLC was similar, demonstrating that the vectors could be feeding either indoors or outdoors. This trend and the considerable indoor resting density of the vectors could support a deployment of IRS which targets endophagic and endophilic vector populations. Most vectors of all sites were recorded actively biting between 10:00 p.m. and 3:00 a.m., and this biting period represents the ideal reported biting time for effective vector control using both recommended strategies such as ITNs and IRS [34]. The period of biting activity mostly coinciding with human sleeping time appears to be an important determinant of vector population vulnerability to interventions [13]. Additionally, the monthly biting behaviour of the vector has yielded different biting peaks and infectivity according to sites. Sakassou recorded two high peaks compared to the others including two peaks of EIRs, which could be related to the particularly irrigated rice field activities undertaken in the area, while the other sites biting, and infection peaks coincided with the rainy season during the year. If rain has a specific factor on increasing larval habitats and mosquito density, it is also known that rice paddies contribute largely to mosquito proliferation, infection, and transmission [35–37]. The trends have always been a particular focus for vector control interventions including social and human behaviour change, and communication to protect the population. In such periods, some of the countries intensify their vector control approaches and strategies by adding complementary measures such as seasonal malaria chemotherapy, larval source management, or intensified sensitization campaigns [38–40].

Furthermore, insecticide susceptibility test using pirimiphos-methyl and clothianidin that indicated susceptibility to pirimiphos-methyl and clothianidin in 11 and 9 of the 17 tested sites respectively, including Sakassou and Nassian, provided options for IRS sprayable insecticides in the country.

### Stratified mass ITN distribution decision making

**Fig. 9:**
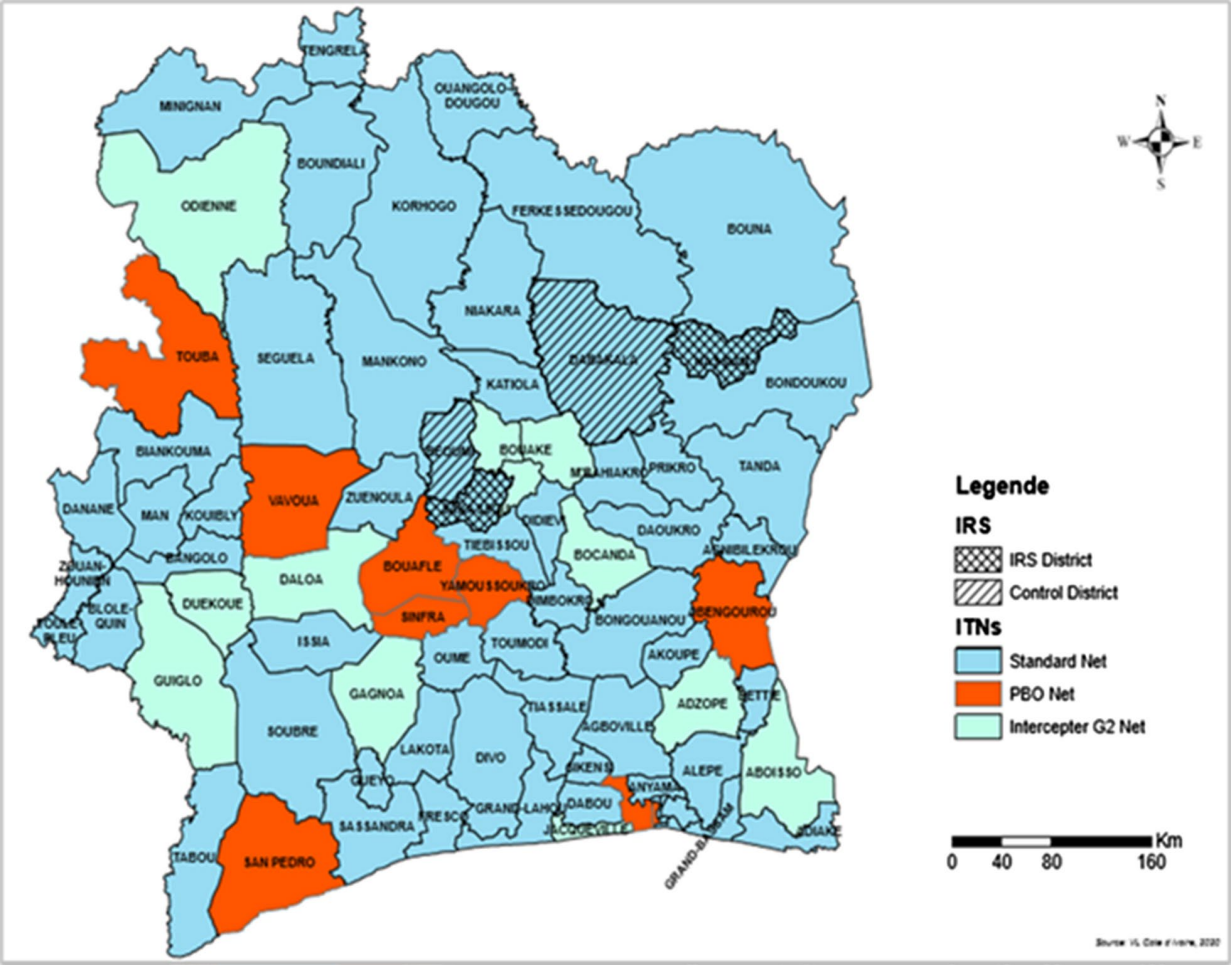
Map of stratified ITN mass distribution and IRS sites in Côte d’Ivoire

The first plan was thus made for the different sites where the resistance status of the vector populations was available and then extrapolated to the other districts in order to cover all 113 health districts within the country. Consequently, the availability of updated data supported the inclusion of Interceptor G2 and PBO ITNs in targeted districts where the susceptibility to chlorfenapyr or significant increase in mortality using PBO was observed. Standard nets treated with alpha-cypermethrin and deltamethrin were further considered as secondary options per resource constraints but using the vector profile of each site to be targeted with either of each insecticide (Fig. 9). Eleven districts were, therefore, covered with PBO-ITNs, eighteen districts with Interceptor G2 and the remaining districts receiving either deltamethrin (75 districts) or alpha-cypermethrin (9 districts)-based ITNs considering the resistance profile against both insecticides at each district. Even though the pyrethroid-only ITNs still show minimum protection against malaria vectors [41] and no concrete ITN failure threshold has been set to date, several studies have reported the added value of new types of ITNs and particularly in area of high insecticide resistance of the mosquitoes [42–44]. Further consideration will need to be taken for all upcoming mass distribution campaigns to enable coverage of the whole country’s households with new generations of ITNs to help mitigate insecticide resistance.

Finally, for the 113 health districts that composed the country with varying resistance patterns described, the NMCP was able to purchase about 20 million of ITNs to ensure the entire 32,172,759 people of Côte d’Ivoire received the most appropriate ITNs for the upcoming campaign, including Interceptor G2 and PBO ITNs.

### IRS district selection and implementation decision making

As IRS is still not widely achievable in endemic countries due to budgetary factors, several factors need to be considered before conducting IRS, including (i) the choice of the appropriate and targeted districts in-country, dependent on malaria incidence and the country’s elimination goals, (ii) the targeted district vector population density and behaviour, (iii) the insecticide resistance status of the vectors to IRS insecticide, (iv) the periodic malaria incidence data, and (v) financial resources, accessibility, and security. In Côte d’Ivoire, the selection of districts to be targeted for IRS was first supported by the epidemiological data provided by each district. Furthermore, the NMCP prioritized districts presenting the highest malaria incidence across the country for implementing IRS considering its goal to decrease transmission in highly endemic districts. In addition, following the available resources and the constraints of IRS operation, other parameters like the size of the district in terms of population, number of structures and the geographical accessibility have been considered in IRS site selection. But susceptibility of local vectors to the available IRS insecticides and vector biting and resting behaviours remained the determinant parameters for appropriate IRS site selection. With the level of resistance occurring in the country, limited insecticide classes were available for spraying. As encountered in several countries, pyrethroid and carbamate IRS insecticides report short term residual efficacy compared to organophosphate insecticides (pirimiphos-methyl/Actellic®) [45–49]. Furthermore, it becomes crucial to have alternative insecticide molecules for IRS to enable rotation of insecticides for resistance management as pirimiphos-methyl resistance is already being reported in several countries including Côte d’Ivoire [50]. Thus, the use of the new WHO Pre-Qualified clothianidin based insecticides which were reported to have slightly longer residual efficacy turned out to be the applicable alternative to the country [51]. Hence susceptibility to clothianidin, recorded against the local vector populations in Sakassou and Nassian compared to Bettié, has guided the selection of clothianidin-based insecticides to be sprayed in both targeted districts.

With its extremely high mean biting rates (170.1 b/p/n) coupled with the highest reported malaria incidence in the country [52], Sakassou represented the first targeted IRS district in Côte d’Ivoire. Furthermore, Nassian recorded the highest infection rate and reported the second highest malaria incidence of the country in 2018–2019, though the district recorded the lowest density over the collection period. Nassian showed a seasonal vector density and transmission with specific peak density and incidence recorded between September and October, coinciding with the single rainy season in the area. Beoumi and Dabakala recorded slightly similar transmission parameters to Nassian with specific biting and infection peaks around the rainy season. The trends observed, the geographical position and similarity have allowed the selection of Beoumi and Dabakala as control sites for an evaluation of IRS in Sakassou and Nassian, respectively. Moreover, the WHO recommends scheduling IRS application to coincide with the build-up of vector populations just before the onset of the peak transmission season [5]. Even though the epidemiological data reported before the implementation of IRS did not ultimately overlap with the EIRs in Sakassou and Nassian, the timing of IRS and residual efficacy of the WHO recommended IRS insecticides reported in different countries could justify the impact of the intervention during both epidemiological and entomological parameter’s peak periods, As April through August represents the long rainy season in Sakassou while June through September represents the single rainy season in Nassian, the IRS timing was selected, considering all above factors and results. Therefore, the highest vector densities recorded, and the infectivity trends guided IRS campaign planning between April and May in Sakassou, right before the peak of transmission in May, and in June through July in Nassian, right before the peak of infectivity.

In addition, both clothianidin-based products were considered suitable, with Fludora Fusion and SumiShield selected to be sprayed in Sakassou and Nassian respectively.

## Conclusion

Selection of appropriate public health interventions should be based on evidence through entomological monitoring to achieve expected goals. The data collection and analysis conducted prior to the deployment of vector control tools in Côte d’Ivoire guided the selection and implementation of appropriate vector control tools and strategies, with the introduction of new generation of ITNs and IRS. Use of data to drive vector control decision-making is essential for appropriate and targeted interventions. Furthermore, continuous monitoring of intervention effects where possible and consideration for other vector borne diseases, when possible, are required. For both IRS selected districts, the presence of different species in Nassian (*An. gambiae s.s*.) and Sakassou (*An. coluzzii*) will be important to monitor in order to estimate any change in the species population and/or vector behaviour that could affect the introduction of IRS, inform future interventions, and impact assessment.

## Supplementary Information

**Additional file 1:** Data collected, analyses and graphs.

## Data Availability

The datasets used and/or analysed during the current study are available in the Additional file 1 and could also be provided by the corresponding author on reasonable request.
