# Correction: Entomological monitoring data driving decision-making for appropriate and sustainable malaria vector control in Côte d’Ivoire

**DOI:** 10.1186/s12936-023-04462-0

**Published:** 2023-01-27

**Authors:** Bernard Loukou Kouassi, Constant Edi, Allassane Foungoye Ouattara, Armand Kouassi Ekra, Louise Golou Bellai, Janice Gouaméné, Yves Alain Kadio Kacou, Jackson Koffi Ives Kouamé, Armel Hermann Obo Béké, Firmain N’Dri Yokoli, Constant Guy N’Guessan Gbalegba, Emmanuel Tia, Roseline Monsan Yapo, Lucien Yao Konan, Roméo N’Tamon N’Tamon, Maurice Adja Akré, Alphonsine Amanan Koffi, Antoine Mea Tanoh, Pascal Zinzindohoué, Blaise Kouadio, Patricia L. Yepassis Zembrou, Allison Belemvire, Seth R. Irish, Ndombour Gning Cissé, Cecilia Flatley, Joseph Chabi

**Affiliations:** 1 PMI VectorLink project, Abidjan, Côte d’Ivoire; 2 Swiss Centre of Scientific Research in Côte d’Ivoire, Abidjan, Côte d’Ivoire; 3 Centre of Veterinary and Medical Entomology, Abidjan, Côte d’Ivoire; 4 National Institute of Public Hygiene, Abidjan, Côte d’Ivoire; 5 National Institute of Public Health/Pierre Richet Institute, Bouake, Côte d’Ivoire; 6 National Malaria Control Programme, Abidjan, Côte d’Ivoire; 7 U.S. President’s Malaria Initiative, USAID, Abidjan, Côte d’Ivoire; 8 U.S. President’s Malaria Initiative, Centers for Disease Control and Prevention (CDC), Abidjan, Côte d’Ivoire; 9 U.S. President’s Malaria Initiative, USAID, Washington, DC USA; 10 U.S. President’s Malaria Initiative, Entomology Branch, U.S. Centers for Disease Control and Prevention (CDC), Atlanta, GA USA; 11 PMI VectorLink Project, Washington, DC USA

**Correction: Malaria Journal (2023) 22:14** **https://doi.org/10.1186/s12936-023-04439-z**

Following publication of the original article [1], the authors flagged that the legends of Figs. 2 and 3 had been swapped in error. The article has since been updated to correct this.

The authors thank you for reading and apologize for any inconvenience caused.
